# A cautionary perspective regarding the isolation and serial propagation of SARS-CoV-2 in Vero cells

**DOI:** 10.1038/s41541-021-00346-z

**Published:** 2021-06-17

**Authors:** Simon G. P. Funnell, Babak Afrough, John James Baczenas, Neil Berry, Kevin R. Bewley, Rebecca Bradford, Clint Florence, Yann Le Duff, Mark Lewis, Ryan V. Moriarty, Shelby L. O. Connor, Karen L. Osman, Steven Pullan, Sujatha Rashid, Kevin S. Richards, Kimberly J. Stemple, Ivana Knezevic

**Affiliations:** 1 PHE Porton Down, National Infection Service, Public Health England, Salisbury, Wiltshire UK; 2 Quadram Institute Bioscience, Norwich Research Park, Norfolk, UK; 3 World Health Organization, Geneva, Switzerland; 4 Wisconsin National Primate Research Center, University of Wisconsin-Madison, Madison, WI USA; 5 Division of Infectious Disease Diagnostics, National Institute for Biological Standards and Control, London, UK; 6 Biodefense and Emerging Infections Resources, NIH, Bethesda, MD USA; 7 Office of Biodefense, Research Resources and Translational Research, Division of Microbiology and Infectious Diseases, NIAID, NIH, Bethesda, MD USA; 8 Faculty of Health and Life Sciences, Oxford Brookes University, Oxford, UK

**Keywords:** Vaccines, Virology, SARS-CoV-2

## Abstract

An array of SARS-CoV-2 virus variants have been isolated, propagated and used in in vitro assays, in vivo animal studies and human clinical trials. Observations of working stocks of SARS-CoV-2 suggest that sequential propagation in Vero cells leads to critical changes in the region of the furin cleavage site, which significantly reduce the value of the working stock for critical research studies. Serially propagating SARS-CoV-2 in Vero E6 cells leads to rapid increases in genetic variants while propagation in other cell lines (e.g. Vero/hSLAM) appears to mitigate this risk thereby improving the overall genetic stability of working stocks. From these observations, investigators are urged to monitor genetic variants carefully when propagating SARS-CoV-2 in Vero cells.

## Introduction

In light of the rapid development of coronavirus disease 2019 (COVID-19) vaccines that involves the use of severe acute respiratory syndrome coronavirus 2 (SARS-CoV-2) viral stocks for vaccine development, animal studies and human clinical trials, the World Health Organization (WHO) initiated an investigation of the serial propagation of the virus in Vero cells to explore the potential impact of cell substrates on the genetic stability of SARS-CoV-2 virus. The results may have an impact on the interpretation of the data from animal studies as well as from clinical trials and may help to improve the quality and control of viral working stocks used in vaccine development, production and evaluation.

An array of SARS-CoV-2 virus variants have been isolated, propagated and used in in vitro assays, in vivo animal studies and human clinical trials. Ensuring the genetic stability of SARS-CoV-2 during in vitro propagation is essential but has been too frequently overlooked. Our observations of working stocks of SARS-CoV-2 suggest that sequential propagation in Vero cells leads to critical changes in the region of the furin cleavage site (FCS), which significantly reduce the value of the working stock for critical research studies, vaccine development, production, evaluation and use.

## Results

### FSC mutations

One feature of SARS-CoV-2, compared to SARS-CoV, is the presence of a four-amino acid insertion corresponding to the S1/S2 FCS in the spike protein.^1^ This amino acid sequence is necessary for SARS-CoV-2 to replicate in lung epithelial cells but is not required for replication in Vero cells. Further, it has been reported that mutations that change or delete this putative FCS may result in a very prominent phenotypic change in plaque assays.^2,3^ Appropriate selection of host cells for virus propagation is therefore critical to ensure that nucleotide sequences, such as those coding for the FCS, remain identical to those in the original isolate and that the working stock retains pathogenicity similar to that of the original isolate.

The authors of this paper, members of the WHO working group on SARS-CoV-2 virus propagation in cell lines, have pooled the results of carefully analysed genetic data generated from sequencing multiple isolates of serially propagated SARS-CoV-2 in different cell types. Serially propagating SARS-CoV-2 in Vero E6 cells leads to rapid increases in genetic variants, particularly those located in the sequence coding for the FCS of the spike protein. Propagation in other cell lines (e.g. Vero/hSLAM) appears to mitigate this risk by limiting the accumulation of such nucleotide variants thereby improving the overall genetic stability of working stocks. From these observations, we strongly urge investigators to monitor genetic variants carefully when propagating SARS-CoV-2 in Vero cells.

### Early findings

In the early phase of the global response to SARS-CoV-2, quality assurance measures taken by Public Health England (PHE) to check the England 02 isolate provided to the Biodefense and Emerging Infections Resources (BEI Resources) included deep sequencing of the second Vero E6 cell line passage of this isolate. This analysis indicated that, although the first passage (P1) stock had no detectable changes (<1%, EPI_ISL_40703) (Table 1), over 90% of the virus content of the P2 stock and 100% of the P3 stock (multiplicity of infection (MOI) ranging from 1.0E−02 to 1.0E−03) contained a 24-nucleotide in-frame deletion in the spike region resulting in loss of 8 amino acids including the FCS (see details in Supplementary Table 1). This observation raised concern among virologists that SARS-CoV-2 isolates being propagated and studied around the globe were not accurately representing the virus circulating in humans. Efforts were then made to produce new P2 stocks of SARS-CoV-2 England 02, from the P1 Vero stock, without FCS defects (Table 1). The deletion was present in P2 virus stocks grown in Calu-1, Calu-3 and Vero/hSLAM but not at a sufficiently high level to be reported in the consensus as a deletion or mixed signal. Manual inspection of the variant file was carried out for each stock sample to identify the deletion present in lower levels of reads. Illumina whole-genome sequencing at read depths of at least 100 was required for accurate assessment of the emergence of subpopulations of SARS-CoV-2 containing small deletions and substitutions. In addition, we noted that a P2 stock propagated in Calu-1 cells did not lose these variants when grown in Vero/SLAM cells but seemed to retain low levels during propagation, whereas the levels of FCS variants rose rapidly in Vero E6 cells irrespective of their source (Table 1).

**Table 1:** Passage history and FCS deletion levels of England 02 stocks.

Cell line used (source and reference)	Passage number					
	1	2	3	4	5	6
Vero E6 (ECACC, 85020206)	<1%^d^					
Vero E6 (BEI, NR-596)^a^		93%	100%			
Vero E6 (BEI, NR-596)^a^		73%				
Vero\hSLAM (ECACC, 04091501)^a^		1.2%				
Calu-3 (ATCC, HTB55)^a^		<1%^d^				
Calu-1 (ECACC, 93120818)^a^		<1%^d^				
Vero E6 (BEI, NR-596)^b^			28%	54%	80%	
Vero E6 (ECACC, 85020206)^b^			40%	64%	80%	
Vero\hSLAM (ECACC, 04091501)^b^			8.7%	4.3%	13.1%	
Calu-1 (ECACC, 93120818)^a^		4.5%	7.5%			
Vero E6 (ECACC, 85020206)^c^				28%	80%	96%

^a^Derived from the P1 stock propagated in Vero E6 (ECACC, 85020206).

^b^All derived from the P2 stock propagated in Calu-1 cells.

^c^Derived from the P3 propagated in Calu-1 cells.

^d^No deletions detected in over 99% of the reads spanning the FCS region.

Our review of SARS-CoV-2 animal infection studies suggests that some may have been compromised by the use of working stocks containing a large proportion of point mutations within, or deletions of, the FCS. The biological consequences of these genetic changes include both limited infectivity of mammalian model hosts and reduced weight loss in infected hamsters. Control groups infected with the affected virus stocks were noted to develop only low viral loads in samples of respiratory secretions. Here we provide examples that justify the necessity for whole-genome sequencing of propagated SARS-CoV-2 stocks with a high-depth and high-accuracy platform (e.g. Illumina). An analysis of low-frequency minor variants of each virus preparation using a tool such as Lofreq^4^ version 2.1.8 is also suggested as best practice.

In February 2020, the Doherty Institute in Melbourne, Australia supplied PHE with a primary stock of SARS-CoV-2 Victoria 01^5^, which was isolated in Vero/hSLAM cells. PHE continued to propagate Victoria 01 in Vero/hSLAM cells for all subsequent studies (MOI ranging from 2.5E−04 to 1.0E−03), including all in vivo model development and vaccine, drug and therapeutic assessment studies. Illumina deep sequencing of all four of the sequential batches of stocks derived from the Victoria 01 strain and propagated in Vero/hSLAM cells found no detectable FCS deletions (Table 2, see detailed analysis in Supplementary Table 2). In addition, no other mutations (consensus and SNVs) were seen outside of the FCS in other parts of the genome. Hamsters exposed to these SARS-CoV-2 challenge stocks lost up to 20% of body weight after exposure and high viral loads were also seen in hamsters, ferrets and primates in samples of mucosal secretions when this stock was used as an inoculum for in vivo studies (Table 2).

**Table 2:** Passage history, FCS deletion levels and in vivo outcomes of Victoria 01 stocks.

Cell line used	Passage number				Model species		
	1	2	3	4	Hamster	Ferret	Primates
Vero/hSLAM	<1%^a^	<1%^a^	<1%^a^	<1%^a^	10–20% weight loss^b^	High viral loads^c^	High viral loads^c^

^a^No deletions detected in over 99% of the reads spanning the FCS region.

^b^National Institute for Biological Standards and Control (NIBSC) and PHE data independently obtain this outcome.

^c^For the purposes of this article, high viral load is characterized as viral RNA titres at or above 1.0E+07 RNA copies/ml in nasal passage or lung samples and low viral load is characterized as below that concentration.

### Collaborative findings

Since February 2020, the NIAID BEI Resources programme has made 28 strains of SARS-CoV-2 available. These stocks were passaged in mammalian cell culture, including Vero (CCL-81^6^) and Vero E6 cells (CRL-1586). Illumina deep sequencing of the early passage stocks demonstrated that <1% of the sequences from P1, P2 and P3 (MOI ranging from 1.0E−03 to 5.0E−03) contained mutations within the FCS (Table 3, see details in Supplementary Table 3).

**Table 3:** Passage history, FCS deletion levels and in vivo outcomes of Washington 01 stocks.

Cell line used	Passage number					Model species	
	1	2	3	4	5	Hamster	Primate
Vero (CCL-81)	<1%^a^	<1%^a^	<1%^a^				
Vero E6 (CRL-1586)				<1%^a,b^	10–18%^d^	10–20% weight loss	Low viral load
Vero E6 (CRL-1586)				<16^c^	58–78%	No weight loss	Low viral load

^a^No deletions detected in over 99% of the reads spanning the FCS region.

^b^This P4 stock is identified by BEI as NR-52281, Lot 70033175.

^c^This P4 stock is identified by BEI as NR-52281, Lot 70036318.

^d^Vero propagated challenge stock referenced in the text.

BIOQUAL Inc. received two separate lots of the described P4 stock of the Washington strain SARS-CoV-2 seed stock from BEI Resources. Lot no. 70033175 (received February 2020) and Lot no. 70036318 (received June 2020) were each expanded using the same conditions as BEI (MOI ranging from 1.0E−03 to 5.0E−03 in Vero E6 cells) at BIOQUAL into three different P5 challenge stocks. Viral RNA was isolated from all six stocks and sequenced by Illumina. In all of the P5 stocks, at least 10% and up to 21% of the sequences of the individual batches exhibited the same single-nucleotide polymorphism (SNP) mutation of the FCS seen in Lot no. 70036318. In addition, all of the P5 stocks derived from Lot no. 70036318 contained between 58 and 78% of reads containing a deletion between locations 23,594 and 23,629 as well as other SNPs in the spike protein (Supplementary Table 3) and other regions (Supplementary Table 4). The variants were almost exclusively present in the spike gene, and three of them directly affected the FCS. Two variants were missense mutations within the FCS, and the third, more common variant, was a 36-nucleotide deletion that eliminated 12 amino acids within the FCS.

Three P5 working stocks were produced from NR-52281 (Lot 70033175) using Vero cells, all of which were found to contain both substitutions (including G23607T [mean 10.9%], C23606T [mean 6.9%] and other SNPs) and deletion (23,583–23,597 deletion [mean 12%]) in the spike region (Supplementary Table S3, lines 3–6) along with other changes present in the seed stock (Supplementary Table S4). One of these P5 stocks (Table S3, line 4), when used as an inoculum, induced 10−20% weight loss in hamsters (Table 3). In addition, this same working stock (marked as superscript “d” in Table 3) was used as an inoculum in a primate infection study (Corbett et al.^7^). Samples taken from the lung and nasal passage of untreated rhesus macaques between days 1 and 4 post-challenge had low virus titres and genetic analysis suggests that the proportion of inoculum containing these FCS deletions and SNPs was not successful in propagating in vivo.

In addition, hamsters infected with a passage 5 stock derived from Lot no. 70036318 (Table 3, line 3) had little or no weight loss. This suggests that the effective dose of a SARS-CoV-2 challenge stock containing a large proportion of FCS defects is substantially lower than that predicted from virus titration in Vero E6 cells.

## Discussion

Studies conducted at PHE, NIBSC, University of Wisconsin-Madison and BEI Resources all conclude that, when SARS-CoV-2 is propagated in Vero E6 cells, there is a risk that during the sequential passage of this virus for working stock generation, deletions may arise in critical virulence components of the virus, including the FCS. Such deletions appear to result in the stock virus being less virulent in animal models (as measured by clinical observations and/or viral titration in mucosal secretions).

However, Vero/hSLAM cells have been found by these four institutions to reduce the apparent rate of mutant production in stocks of SARS-CoV-2. Consequently, the authors suggest that propagation of SARS-CoV-2 in this cell line will reduce the risk of viral progeny containing defective mutations when producing working stocks.

On the basis of this preliminary data, we encourage researchers producing stocks of SARS-CoV-2 to consider:

- limitation of the number of passages in cell culture, using low MOI, in an effort to maintain wild-type properties;
- evaluation and selection of a cell line that supports viral isolation and working stock production with acceptable (<1%) variant thresholds for downstream use;
- evaluation of both the consensus sequence and inclusion of analysis of minor variants of each virus preparation;
- incorporation of LoFreq^4^ (or equivalent) sequencing analysis for low-frequency variant calling.

Spontaneous mutations due to virus adaptation to both Vero and Vero E6 cells have also been reported for viruses such as Ebola virus and Zika virus.^8,9^ However, deletions and mutations in the SARS-CoV-2 FCS became so frequently observed in passages 4 and 5 that they dominated the reads taken from workings stocks by up to 99% (Tables 1 and 3). The results at passage 4 were, however, variable such that the FCS region of two different passage 4 stock contained mutations at a frequency of ≈16% in one stock but <1% for another (Table 3). This latter stock when taken to passage 5 did, however, yield a stock with >10% FCS variants. These data suggest that even the same passage level of virus can exhibit entirely different genetic characteristics, further emphasizing that investigators need to confirm the genetic sequence after propagation rather than relying on the sequence of the seed stock.

The findings of this group in this publication support the observations of other groups^2,10,11^ that FCS changes occur during serial propagation of SARS-CoV-2 in some cell lines. Despite the publication of these articles, there is continued production and dissemination of stocks of virus that are compromised in this manner, especially as there is a perceived need to rapidly isolate and distribute new variants with a combination of changes in the spike protein. Working stock propagation compromised in this manner will lead to stocks that are not fully able to infect mammalian host species. In such scenarios, control groups will appear to behave as if they have effectively been infected with a smaller dose than that predicted from the live virus titration (in focus forming or plaque forming units) of the challenge inoculum and in subgenomic PCR assessment and live virus load analysis of samples of respiratory secretions or mucosa taken after infection. This will compromise the ability of studies that claim to use the same viral challenge dose because studies using FCS-compromised stocks will effectively have lower challenge doses. Similarly, in assays that assess human sera for their ability to neutralize live virus, the challenge inoculum with not be fully representative of the natural inoculum and will risk generating misleading outcomes in clinical trials and a lack of comparability of studies. Additionally, vaccines that use whole virus should be fully sequenced to ensure that large production batches are not FCS deletion deficient.

WHO recommendations for cell culture production, including principles for good cell culture practice, need to be followed when propagating SARS-CoV-2 virus.^12^ Laboratories are urged to conduct careful genetic analyses of stocks obtained using techniques that have the resolution to detect nucleotide variants and minor deletions in the FCS. Authors and scientific publishers are encouraged to insist on including data in manuscripts that report a detailed genetic analysis of working virus stocks used in each reported study and that the proportion of FCS variants is quantified and reported. This would lead to better understanding of the SARS-CoV-2 virus characteristics used for vaccine development, production and evaluation.

## Methods

### Public Health England (PHE)

The PHE process for extraction, library preparation and sequencing of NGS SARS-CoV-2 England/02/2020 (EPI_ISL_407073) started with a nucleic acid extraction using the QIAamp Viral RNA Kit (Qiagen) eluting in 50 μl of H_2_O. A DNAse step followed with the addition of Turbo DNase (Thermo Fisher Scientific) and incubation at 37 °C for 30 min. The RNA Clean & Concentrator-5 Kit (Zymo Research) was used to purify and concentrate the RNA following the manufacturer’s instructions. Using a sequence-independent single-primer amplification method, randomly amplified complementary DNA (cDNA) was produced for each sample using the A/B method as previously described.^13^ Library preparation was carried out using the NEXTERA XT V2 Kit (Illumina) using 1 ng of the resulting SISPA B product to the manufacturer’s instructions and sequenced on a 2× 150-bp paired-end Illumina MiSeq run by the Genomics Services Development Unit of PHE.

The PHE Bioinformatics analysis started when Raw FASTQ data were mapped to the reference genome EPI_ISL_407073_(England_2) using BWA MEM at default parameters. Samtools converted, sorted and indexed SAM file into required BAM file format. Quasi_bam (PHE in-house application) was used to create a consensus sequence and a variant file. Consensus sequence was called using parameters as follows: minimum mapping quality of 30, minimum depth of 100, SNPs/Indels where present in a minimum of 90% of reads, minor variants (those reported as IUPAC ambiguous codes in consensus sequence) were called where an alternative base was present in ≥20% of reads. The float parameter (the threshold for reporting variance levels in the variants file) was lowered to 0.1% to further investigate the sequence present at the site of the Bristol deletion in more detail. As minor variants are called into the consensus sequence at a conservative 20%, manual inspection of the variant file was carried out for each stock sample to identify the deletion present in lower levels of reads.

### BEI resources

SARS-CoV-2 samples were library prepped using the NEBNext® Ultra II™ RNA Library Prep Kit for Illumina® (NEB #E7775) according to the manufacturer’s protocols. The samples were sequenced on the Illumina MiSeq platform using either a MiSeq® Reagent Micro Kit v2 (300 Cycle) or a MiSeq® Reagent Kit v2 (500 cycle) run for 300 cycles. Reads produced were trimmed using the AMGP readsQC-illumina.py pipeline (comprised of Trimmomatic v0.38 using default parameters and FastQC v0.11.9) to remove low-quality bases and adaptor sequences. Reference-based assembly was performed using the following genomes as a reference sequences from GenBank: MT246667.1 (SARS-CoV-2 strain FDAARGOS_983 isolate SARS-CoV-2/human/USA/USA-WA1/2020, complete genome) and the clinical reference sequence for the interrogated strain.

Reads were then mapped to each reference sequence using minimap2 2.17-r974-dirty in short read mode using the -ax sr flag. Reference regions with <10× mean coverage were considered poorly supported low-coverage regions. Reference coverage statistics were reported for each set of mapped reads.

Analysis was performed by identifying variants relative to the respective reference sequence using bcftools (v.1.10.2) mpileup default parameters, except for an increased maximum depth parameter (-d) of 8000, and bcftools call with default parameters. Variants with quality scores falling below 30 (Phred-scaled) were filtering using bcftools filter, then normalized using bcftools norm with default parameters. The consensus sequence was generated using bcftools by iteratively applying alternative alleles at >95% frequency to the reference, followed by applying alternative alleles between >5% and ≤95% as ambiguous bases and masking low-coverage regions as N as reported by bedtools genomecov. Flanking low coverage regions were removed. Variant summary statistics were reported for each strain.

In order to maintain consistent reporting of nucleotide location for purposes of comparison and analysis, all variants are reported in the standard format globally, i.e. relative to the sequence location within the SARS-CoV-2 Wuhan-Hu-1 reference genome (NC_045512.2). For a nucleotide at Position *X*, the variant frequency is calculated by: Variant frequency = (# reads with variant nucleotide mapped to Position *X*)/(total # reads mapped to Position *X*)

BEI Resources recently incorporated Lofreq version 2.1.8 according to its suggested best practices, which include

1) switching to using BWA‐MEM as the primary read aligner,

2) implementing a read realignment step via the Viterbi algorithm (an optional command provided as part of Lofreq),

3) implementing GATK’s machine learning approach to base quality recalibration (GATK BQSR) on the read alignment.

GATK BQSR requires an initial variant call set to calibrate base quality scores; to perform this initial variant calling, after read realignment via the Viterbi algorithm, the variants are called using bcftools and then fed into GATK BQSR to correct the read alignment, and then call final variants using Lofreq from the corrected alignment. Initial results from this evaluation show a lower limit of detection of variants with the caveat that Lofreq pipeline tended to produce spurious variants (i.e. false positives) at frequencies <5%. We applied this modified pipeline to previously analysed BEI Resources SARS-CoV-2 read sets and detected all existing variants and found additional variants at frequencies ≤25%. We also ran collaborator read sets on this pipeline and find substantial agreement between BEI Resources detected variants and collaborator variant calls.

### University of Wisconsin—Madison (UWM)

For sample preparation and sequencing for SARS-CoV-2 genomes at UWM, SARS-CoV-2 inocula were sequenced using a modified approach originally developed by ARTIC Network. Briefly, cDNA was synthesized using SuperScript IV Reverse Transcriptase (Invitrogen, Carlsbad, CA, USA), random hexamers and dNTPs. The cDNA was then PCR-amplified following a multiplex PCR amplicon-based approach that was developed for Nanopore. Two multiplexed PCR reactions (with a total of 96 primers) were performed with Q5 Hot Start Hi-Fi 2x Master mix (New England Biolabs, Ipswich, MA, USA) and cDNA as the starting template. The following thermocycling conditions were used: 98 °C for 30 s, followed by 25 cycles of 98 °C for 15 s and 65 °C for 5 min, then followed by an indefinite hold at 4 °C. The amplified products were pooled together and purified with AMPure XP beads (Beckman Coulter, Brea, CA, USA). Libraries were prepared with the TruSeq Sample Preparation Kit (Illumina, USA). Samples were end repaired and then purified using the Sample Purification Beads (SPB). Each sample was then A-Tailed by attaching a non-templated nucleotide to the 3’ end, followed by an adaptor ligation phase. A post ligation bead cleanup using SPB was performed. Finally, each sample was amplified via eight cycles of PCR, followed by a bead cleanup using SPB, and eluted in RSB. The concentration and average fragment length were determined with a Qubit dsDNA High-Sensitivity Kit (Invitrogen, USA) and Agilent’s High Sensitivity DNA Kit, respectively. Each sample was pooled equimolarly to a concentration of 4 nM. This pool was denatured with 5 μl of 0.2 N NaOH, vortexed and incubated at room temperature for 5 min. HT1 buffer solution was added to generate a 20-pM pool. The 20-pM pool was then diluted to a final concentration of 10 pM and a Phix-derived control was spiked in, to account for 10% of the total DNA. The pool was loaded onto a 2 × 250 cycle V2 cartridge, to be sequenced on an Illumina MiSeq.

For bioinformatics analysis of raw sequencing of data at UWM, an analytical pipeline called “Zequencer V7” was used to process the raw FASTQ files. In short, the primer sequences were trimmed and the reads were paired and merged using BBDuk (https://jgi.doe.gov/data-and-tools/bbtools/bb-tools-user-guide/bbduk-guide/) and BBMerge (https://jgi.doe.gov/data-and-tools/bbtools/bb-tools-user-guide/bbmerge-guide/). The reads were then mapped to the reference (MN908947.3) using BBMap (https://jgi.doe.gov/data-and-tools/bbtools/bb-tools-user-guide/bbmap-guide/). The BAM alignments were imported into Geneious Prime 2021.1.1, and variants were called at a threshold of 1% (https://www.geneious.com). The entire Zequencer analysis pipeline is available at https://github.com/DABAKER165/zequencer_ncov19.

### Reporting summary

Further information on research design is available in the Nature Research Reporting Summary linked to this article.

## Supplementary information

Supplementary Information

Reporting Summary

## Data Availability

SARS-CoV-2 sequence data that support the findings of this study have been deposited in “SRA” and “GenBank” with the accession codes as detailed in Supplementary Table 5.

## Code availability

No previously unreported custom computer code or algorithms were used to generate results that are reported in the paper and central to its main claims.

## References

[1] Hoffmann M, Kleine-Weber H, Pohlmann S (2020). A multibasic cleavage site in the spike protein of SARS-CoV-2 is essential for infection of human lung cells. Mol. Cell.

[2] Davidson AD (2020). Characterisation of the transcriptome and proteome of SARS-CoV-2 reveals a cell passage induced in-frame deletion of the furin-like cleavage site from the spike glycoprotein. Genome Med..

[3] Ogando NS (2020). SARS-coronavirus-2 replication in Vero E6 cells: replication kinetics, rapid adaptation and cytopathology. J. Gen. Virol..

[4] Wilm A (2012). LoFreq: a sequence-quality aware, ultra-sensitive variant caller for uncovering cell-population heterogeneity from high-throughput sequencing datasets. Nucleic Acids Res..

[5] Caly L (2020). Isolation and rapid sharing of the 2019 novel coronavirus (SARS-CoV-2) from the first patient diagnosed with COVID-19 in Australia. Med. J. Aust..

[6] Harcourt J (2020). Severe acute respiratory syndrome coronavirus 2 from patient with coronavirus disease, United States. Emerg. Infect. Dis..

[7] Corbett KS (2020). Evaluation of the mRNA-1273 vaccine against SARS-CoV-2 in nonhuman primates. N. Engl. J. Med..

[8] Ruedas JB, Arnold CE, Palacios G, Connor JH (2018). Growth-adaptive mutations in the Ebola virus makona glycoprotein alter different steps in the virus entry pathway. J. Virol..

[9] Duggal NK (2019). Mutations present in a low-passage Zika virus isolate result in attenuated pathogenesis in mice. Virology.

[10] Johnson, B. A. et al. Furin cleavage site is key to SARS-CoV-2 pathogenesis. Preprint at *bioRxiv*10.1101/2020.08.26.268854 (2020)

[11] Lamers, M. M. et al. Human airway cells prevent SARS-CoV-2 multibasic cleavage site cell culture adaptation. *Elife* **10**, e66815 (2021) 10.7554/eLife.66815 PMC8131099 33835028

[12] WHO. Recommendations for the evaluation of animal cell cultures as substrates for the manufacture of biological medicinal products and for the characterization of cell banks. In *WHO Expert Committee on Biological Standardization: Sixty-First Report*. Annex 3 (WHO Technical Report Series, No. 978) (World Health Organization, 2013)

[13] Lewandowski K (2019). Metagenomic nanopore sequencing of influenza virus direct from clinical respiratory samples. J. Clin. Microbiol..

