# Supplementary material for: A cautionary perspective regarding the isolation and serial propagation of SARS-CoV-2 in Vero cells
Source: NPJ Vaccines. 2021 Jun 17;6:83. doi: 10.1038/s41541-021-00346-z (PMC8211640; doi:10.1038/s41541-021-00346-z)
Supplement: Supplementary file 1 — Supplementary Information [file 41541_2021_346_MOESM1_ESM.pdf]

Supplementary Table 1. Sequencing statistics for all samples cited in Table 1

			Genome coverage			FCS coverage		
**Sample ID**	**Passage**	**Cell**	**# of Reads**	**% Coverage**	**Mean read depth**	**FCS Deletion**	**Read depth***	**% deletion****
PHE_COVID_Eng02_2171	P1	Vero E6 ECACC	1119074	100.00	4689	None detected	5603	<1
PHE_COVID_Eng02_2175	P2	Vero E6 BEI	2221101	100.00	7185	23598-23621	5991	93.91
PHE_COVID_Eng02_2176	P2	Vero E6 BEI	1572395	100.00	5132	23598-23621	4176	74.89
PHE_COVID_Eng02_2341	P3	Vero E6 BEI	657460	99.93	2115	23598-23621	642	99.60
PHE_COVID_Eng02_3134	P2	Vero/hSLAM	2461490	100.00	7999	23609-23621	11146	1.20
PHE_COVID_Eng02_3144	P2	Calu-3	336962	99.99	1378	None detected	1598	<1
PHE_COVID_Eng02_3139	P2	Calu-1	1252948	99.99	4094	None detected	4622	<1
PHE_COVID_Eng02_3541	P2+1 (Calu-1 P1-P2)	Vero E6 BEI	2997648	99.99	9767	23598-23621	12190	27.71
PHE_COVID_Eng02_3568	P2+1 (Calu-1 P1-P2)	Vero E6 BEI	15548981	99.99	50940	23598-23621	14858	54.31
PHE_COVID_Eng02_3586	P2+1 (Calu-1 P1-P2)	Vero E6 BEI	4289539	99.99	13921	23598-23621	45795	80.15
PHE_COVID_Eng02_3540	P2+1 (Calu-1 P1-P2)	Vero E6 ECACC	1672355	100.00	7220	23598-23621	11188	40.19
PHE_COVID_Eng02_3567	P2+1 (Calu-1 P1-P2)	Vero E6 ECACC	5273199	100.00	17193	23598-23621	18153	64.18
PHE_COVID_Eng02_3585	P2+1 (Calu-1 P1-P2)	Vero E6 ECACC	4433547	100.00	14470	23598-23621	13567	80.15
PHE_COVID_Eng02_3254	P2	Calu-1	3443497	99.99	11310	23598-23621	10690	4.54
PHE_COVID_Eng02_3468	P3	Calu-1	1886612	99.99	6192	23598-23621	8987	7.51
PHE_COVID_Eng02_3466	P3	Vero/hSLAM	3449103	99.99	11314	23598-23621	11609	8.72
PHE_COVID_Eng02_3470	P4	Vero/hSLAM	4269861	99.99	14018	23598-23621	14034	5.36
PHE_COVID_Eng02_19_65_4xC0	P5	Vero/hSLAM	759752	99.99	2828	23598-23621	2870	13.18
PHE_COVID_Eng02_H7_168h	P3+1 (Vero/hSLAM P1-P3)	Vero E6 ECACC	2791855	99.99	9137	23598-23621	10715	28.39
PHE_COVID_Eng02_H7_336h	P3+1 (Vero/hSLAM P1-P3)	Vero E6 ECACC	1086387	99.99	4325	23598-23621	5437	80.52
PHE_COVID_Eng02_H7_504h	P3+1 (Vero/hSLAM P1-P3)	Vero E6 ECACC	619718	100.00	2395	23598-23621	2619	95.29

* Average read depth across area of deletion (or 23598-23621bp if no deletion)
** Average percentage of reads containing deletion across sequence.

Supplementary Table 2. Sequencing statistics for all samples cited in Table 2

			Genome coverage			FCS coverage		
**Sample ID**	**Passage**	**Cell**	**# of Reads**	**% Coverage**	**Mean read depth**	**FCS Deletion**	**Read depth***	**% deletion****
PHE_COVID_Vic01_ASL349	P3	Vero/hSLAM	2545422	100.00	8333.00	No	9514.96	<1%
PHE_COVID_Vic01_ASL405	P3	Vero/hSLAM	1746564	100.00	5600.99	No	7586.71	<1%
PHE_COVID_Vic01_ASL415	P4	Vero/hSLAM	3258235	100.00	10662.70	No	1845.58	<1%
PHE_COVID_Vic01_ASL427	P4	Vero/hSLAM	4004065	100.00	13121.90	No	18474.61	<1%

* Average read depth across area of deletion (or 23598-23621bp if no deletion)

** Average percentage of reads containing deletion across sequence.

Supplementary Table 3. Sequencing statistics for all samples cited in Table 3

			Genome coverage			FCS coverage (and percentage deletions or SNPs**)								
**Sample ID**	**Passage**	**Cell**	**# of Reads**	**% Coverage**	**Mean read depth**	**FCS Deletions**	**Read depth***	**%23583-23597 del**	**%23598-23607 del**	**%23594-23629 del**	**%23627-23635 del**	**% C23606T**	**% G23607A**	**% G23607T**
BEI NR-52281 Lot 70033175	P4	Vero E6 (CRL-1586)	3590997	99.9966	28221	None detected	9476	<1	<1	<1	<1	<1	<1	<1
BEI NR-52281 Lot 70036318	P4	Vero E6 (CRL-1586)	293017	99.9632	1372	23606	1785	<1	<1	<1	<1	9.17	<1	15.93
Seed_3175_Stock_0223_R1	P5	Vero E6	442647	99.90	5079	23583-23597	3924	10.00	<1	<1	<1	3.5	N/A	10.4
Seed_3175_Stock_0427_R2	P5	Vero E6	385859	99.90	4453	23583-23598	2903	2.50	<1	<1	<1	2.8	1.2	10.6
Seed_3175_Stock_0616_R1	P5	Vero E6	604620	99.80	7084	Multiple sites	3686	46.60	2.45	<1	7.10	4.4	2.7	18.2
Seed_3175_Stock_0616_R2	P5	Vero E6	603175	100.00	6988	Multiple sites	3994	48.10	2.45	<1	8.30	3.9	2.8	16.7
Seed_6318_Stock_7142_R1	P5	Vero E6	593783	100.00	6974	Multiple sites	2293	1.70	<1	77.80	<1	7.2	1.1	10.1
Seed_6318_Stock_7142_R2	P5	Vero E6	382907	100.00	4392	Multiple sites	3541	2.00	<1	77.30	<1	7.1	1.3	10.3
Seed_6318_Stock_7152_R1	P5	Vero E6	604816	99.90	7014	Multiple sites	3603	2.00	<1	70.00	<1	9.7	1.2	14.7
Seed_6318_Stock_7152_R2	P5	Vero E6	424033	99.90	4832	Multiple sites	3571	2.30	<1	69.80	<1	9.1	1.3	15.2
Seed_6318_Stock_0825_R1	P5	Vero E6	598679	100.00	7006	Multiple sites	2611	2.70	<1	58.30	<1	11.3	1.7	20.7
Seed_6318_Stock_0825_R2	P5	Vero E6	599783	99.90	6993	Multiple sites	4887	2.30	<1	60.30	<1	10	1.6	21.4

* Average read depth across area of deletion (or 23598-23621bp if no deletion)
Nucleotides 23603 to 23617 spans the PRRAR sequence

** Average percentage of reads containing deletion or SNPs across sequence.

Supplementary Table 4. Other notable variations in BEI Lots of stock NR-52281

BEI NR-52281 Lot 70033175 (FDA ARGOS GenBank MT246667)

Sample ID	Passage	Cell	Type	Variant	Coverage	Quality	Length	Sample Frequency	Gene (Region)	AA Mutation
BEI NR-52281 Lot 70033175	P4	Vero E6 (CRL-1586)	SNP	c8782t	20,170	49,314	1	99.87%	ORF1ab (nsp4)	--
			SNP	c18060t	26,016	49,314	1	99.65%	ORF1ab (nsp14)	--
			SNP	t28144c	81,777	49,314	1	99.70%	ORF8	L84S

BEI, NR-52281, Lot 70036318 (GenBank MW811435)

Sample ID	Passage	Cell	Type	Variant	Coverage	Quality	Length	Sample Frequency	Gene (Region)	AA Mutation
BEI NR-52281 Lot 70036318	P4	Vero E6 (CRL-1586)	SNP	c8782t	1,264	44,731	1	99.84%	ORF1ab (nsp4)	--
			SNP	c17827a	1,719	4,590	1	12.74%	ORF1ab (nsp13)	Q531K
			SNP	c18060t	1,641	49,314	1	99.88%	ORF1ab (nsp14)	--
			SNP	a22206g	842	780	1	5.45%	S	D215G
			SNP	c22482t	1,473	6,040	1	17.04%	S	T307I
			SNP	c23606t	1,785	2,477	1	9.17%	S	R682W
			SNP	g23607t	1,763	5,155	1	15.93%	S	R682L
			SNP	t28144c	2,584	49,314	1	99.92%	OFR8	L84S
			SNP	a29839g	1,142	435	1	5.10%	3’ UTR	n/a

Supplementary Table 5. Sequence data and links for the samples cited in Tables 1-3

Submitting Institute	Accession	Strain	Link	Repository
Public Health England, Porton Down, UK	SAMN18713629	SARS-CoV-2/human/England/02/2020	https://www.ncbi.nlm.nih.gov/biosample/SAMN18713629	SRA
Public Health England, Porton Down, UK	SAMN18713630	SARS-CoV-2/human/England/02/2020	https://www.ncbi.nlm.nih.gov/biosample/SAMN18713630	SRA
Public Health England, Porton Down, UK	SAMN18713631	SARS-CoV-2/human/England/02/2020	https://www.ncbi.nlm.nih.gov/biosample/SAMN18713631	SRA
Public Health England, Porton Down, UK	SAMN18713632	SARS-CoV-2/human/England/02/2020	https://www.ncbi.nlm.nih.gov/biosample/SAMN18713632	SRA
Public Health England, Porton Down, UK	SAMN18713633	SARS-CoV-2/human/England/02/2020	https://www.ncbi.nlm.nih.gov/biosample/SAMN18713633	SRA
Public Health England, Porton Down, UK	SAMN18713634	SARS-CoV-2/human/England/02/2020	https://www.ncbi.nlm.nih.gov/biosample/SAMN18713634	SRA
Public Health England, Porton Down, UK	SAMN18713635	SARS-CoV-2/human/England/02/2020	https://www.ncbi.nlm.nih.gov/biosample/SAMN18713635	SRA
Public Health England, Porton Down, UK	SAMN18713636	SARS-CoV-2/human/England/02/2020	https://www.ncbi.nlm.nih.gov/biosample/SAMN18713636	SRA
Public Health England, Porton Down, UK	SAMN18713637	SARS-CoV-2/human/England/02/2020	https://www.ncbi.nlm.nih.gov/biosample/SAMN18713637	SRA
Public Health England, Porton Down, UK	SAMN18713638	SARS-CoV-2/human/England/02/2020	https://www.ncbi.nlm.nih.gov/biosample/SAMN18713638	SRA
Public Health England, Porton Down, UK	SAMN18713639	SARS-CoV-2/human/England/02/2020	https://www.ncbi.nlm.nih.gov/biosample/SAMN18713639	SRA
Public Health England, Porton Down, UK	SAMN18713640	SARS-CoV-2/human/England/02/2020	https://www.ncbi.nlm.nih.gov/biosample/SAMN18713640	SRA
Public Health England, Porton Down, UK	SAMN18713641	SARS-CoV-2/human/England/02/2020	https://www.ncbi.nlm.nih.gov/biosample/SAMN18713641	SRA
Public Health England, Porton Down, UK	SAMN18713642	SARS-CoV-2/human/England/02/2020	https://www.ncbi.nlm.nih.gov/biosample/SAMN18713642	SRA
Public Health England, Porton Down, UK	SAMN18713643	SARS-CoV-2/human/England/02/2020	https://www.ncbi.nlm.nih.gov/biosample/SAMN18713643	SRA
Public Health England, Porton Down, UK	SAMN18713646	SARS-CoV-2/human/England/02/2020	https://www.ncbi.nlm.nih.gov/biosample/SAMN18713646	SRA
Public Health England, Porton Down, UK	SAMN18713644	SARS-CoV-2/human/AUS/VIC01/2020	https://www.ncbi.nlm.nih.gov/biosample/SAMN18713644	SRA
Public Health England, Porton Down, UK	SAMN18713645	SARS-CoV-2/human/AUS/VIC01/2020	https://www.ncbi.nlm.nih.gov/biosample/SAMN18713645	SRA
National Institute for Biological Standards and Control, UK	SAMN18632196	SARS-CoV-2/human/AUS/VIC01/2020	https://www.ncbi.nlm.nih.gov/biosample/SAMN18632196	SRA
National Institute for Biological Standards and Control, UK	SAMN18632233	SARS-CoV-2/human/AUS/VIC01/2020	https://www.ncbi.nlm.nih.gov/biosample/SAMN18632233	SRA
University of Winsconsin-Madison, USA	SAMN14693018	SARS-Cov2/human/USA/USA-WA1/2020	https://www.ncbi.nlm.nih.gov/biosample/SAMN14693018	SRA
University of Winsconsin-Madison, USA	SAMN16320573	SARS-Cov2/human/USA/USA-WA1/2020	https://www.ncbi.nlm.nih.gov/biosample/SAMN16320573	SRA
University of Winsconsin-Madison, USA	SAMN16846875	SARS-Cov2/human/USA/USA-WA1/2020	https://www.ncbi.nlm.nih.gov/biosample/SAMN16846875	SRA
University of Winsconsin-Madison, USA	SAMN16846874	SARS-Cov2/human/USA/USA-WA1/2020	https://www.ncbi.nlm.nih.gov/biosample/SAMN16846874	SRA
University of Winsconsin-Madison, USA	SAMN16320580	SARS-Cov2/human/USA/USA-WA1/2020	https://www.ncbi.nlm.nih.gov/biosample/SAMN16320580	SRA
University of Winsconsin-Madison, USA	SAMN16320581	SARS-Cov2/human/USA/USA-WA1/2020	https://www.ncbi.nlm.nih.gov/biosample/SAMN16320581	SRA
University of Winsconsin-Madison, USA	SAMN16320578	SARS-Cov2/human/USA/USA-WA1/2020	https://www.ncbi.nlm.nih.gov/biosample/SAMN16320578	SRA
University of Winsconsin-Madison, USA	SAMN16320579	SARS-Cov2/human/USA/USA-WA1/2020	https://www.ncbi.nlm.nih.gov/biosample/SAMN16320579	SRA
University of Winsconsin-Madison, USA	SAMN16320574	SARS-Cov2/human/USA/USA-WA1/2020	https://www.ncbi.nlm.nih.gov/biosample/SAMN16320574	SRA
University of Winsconsin-Madison, USA	SAMN16320575	SARS-Cov2/human/USA/USA-WA1/2020	https://www.ncbi.nlm.nih.gov/biosample/SAMN16320575	SRA
University of Winsconsin-Madison, USA	SAMN16320582	SARS-Cov2/human/USA/USA-WA1/2020	https://www.ncbi.nlm.nih.gov/biosample/SAMN16320582	SRA
University of Winsconsin-Madison, USA	SAMN16320583	SARS-Cov2/human/USA/USA-WA1/2020	https://www.ncbi.nlm.nih.gov/biosample/SAMN16320583	SRA
National Institute of Allergy and Infectious Disease, USA	MT246667.1	SARS-Cov2/human/USA/USA-WA1/2020	https://www.ncbi.nlm.nih.gov/nuccore/MT246667.1	GenBank
National Institute of Allergy and Infectious Disease, USA	MW811435.1	SARS-Cov2/human/USA/USA-WA1/2020	https://www.ncbi.nlm.nih.gov/nuccore/MW811435.1	GenBank
