# Supplementary material for: A cautionary perspective regarding the isolation and serial propagation of SARS-CoV-2 in Vero cells
Source: NPJ Vaccines. 2021 Jun 17;6:83. doi: 10.1038/s41541-021-00346-z (PMC8211640; doi:10.1038/s41541-021-00346-z)
Supplement: Supplementary file 2 — Reporting Summary [file 41541_2021_346_MOESM2_ESM.pdf]

nature research

Corresponding author(s): Ivana Knezevic

Last updated by author(s): Apr 15, 2021

Reporting Summary

Nature Research wishes to improve the reproducibility of the work that we publish. This form provides structure for consistency and transparency in reporting. For further information on Nature Research policies, see our Editorial Policies and the Editorial Policy Checklist.

Statistics

For all statistical analyses, confirm that the following items are present in the figure legend, table legend, main text, or Methods section.

n/a	Confirmed	
☒	☐	The exact sample size (*n*) for each experimental group/condition, given as a discrete number and unit of measurement
☒	☐	A statement on whether measurements were taken from distinct samples or whether the same sample was measured repeatedly
☒	☐	The statistical test(s) used AND whether they are one- or two-sided<br>*Only common tests should be described solely by name; describe more complex techniques in the Methods section.*
☒	☐	A description of all covariates tested
☒	☐	A description of any assumptions or corrections, such as tests of normality and adjustment for multiple comparisons
☒	☐	A full description of the statistical parameters including central tendency (e.g. means) or other basic estimates (e.g. regression coefficient) AND variation (e.g. standard deviation) or associated estimates of uncertainty (e.g. confidence intervals)
☒	☐	For null hypothesis testing, the test statistic (e.g. *F*, *t*, *r*) with confidence intervals, effect sizes, degrees of freedom and *P* value noted<br>*Give P values as exact values whenever suitable.*
☒	☐	For Bayesian analysis, information on the choice of priors and Markov chain Monte Carlo settings
☒	☐	For hierarchical and complex designs, identification of the appropriate level for tests and full reporting of outcomes
☒	☐	Estimates of effect sizes (e.g. Cohen's *d*, Pearson's *r*), indicating how they were calculated

Our web collection on statistics for biologists contains articles on many of the points above.

Software and code

Policy information about availability of computer code

Data collection	At PHE, Samtools converted, sorted and indexed SAM file into required BAM file format. At BEI, minimap2 2.17-r974-dirty in short read mode using the -ax sr flag. At Uni Wisconsin, An analytical pipeline called "Zequencer V7" was using to process the raw FASTQ files. In short, the primer sequences were trimmed and the reads were paired and merged using BBDuk (https://jgi.doe.gov/data-and-tools/bbtools/bb-tools-user-guide/bbduk-guide/) and BBMerge (https://jgi.doe.gov/data-and-tools/bbtools/bb-tools-user-guide/bbmerge-guide/). The reads were then mapped to the reference (MN908947.3) using BBMap (https://jgi.doe.gov/data-and-tools/bbtools/bb-tools-user-guide/bbmap-guide/).
Data analysis	At PHE, Quasi_bam (PHE in-house application) was used to create a consensus sequence and a variants file. At BEI, Analysis was performed by identifying Variants relative to the respective reference sequence using bcftools (v.1.10.2) mpileup default parameters, except for an increased maximum depth parameter (-d) of 8000, and bcftools call with default parameters. At Uni Wisconsin, The BAM alignments were imported into Geneious Prime 2021.1.1, and variants were called at a threshold of 1% (https://www.geneious.com). The entire Zequencer analysis pipeline is available at https://github.com/DABAKER165/zequencer_ncov19.

For manuscripts utilizing custom algorithms or software that are central to the research but not yet described in published literature, software must be made available to editors and reviewers. We strongly encourage code deposition in a community repository (e.g. GitHub). See the Nature Research guidelines for submitting code & software for further information.

Data

Policy information about availability of data

All manuscripts must include a data availability statement. This statement should provide the following information, where applicable:

- Accession codes, unique identifiers, or web links for publicly available datasets
- A list of figures that have associated raw data
- A description of any restrictions on data availability

Data availability
SARS-CoV-2 sequence data that support the findings of this study have been deposited in [repository name e.g. "GenBank"] with the accession codes as detailed in Supplementary Table 5.

Field-specific reporting

Please select the one below that is the best fit for your research. If you are not sure, read the appropriate sections before making your selection.

☒ Life sciences ☐ Behavioural & social sciences ☐ Ecological, evolutionary & environmental sciences

For a reference copy of the document with all sections, see nature.com/documents/nr-reporting-summary-flat.pdf

Life sciences study design

All studies must disclose on these points even when the disclosure is negative.

Sample size	This report documents the independant analysis of viral working stocks by three different institutions using three different viral stock. These qualitative analyses involved high fidelity bioinformatic assessment
Data exclusions	No data were excluded from the study
Replication	The findings of each of three different institutions all agreed on the outcomes of their independent investigations
Randomization	Randomisation was not utilised for these independent analyses of viral stocks. The analyses were controlled by comparison to reference genomes
Blinding	Blinding was not relevant to these qualitative analyses. The bioinformatic algorithms used at each of the three institutions were not susceptible to operator bias and we have provided all of the raw data for independent verification if required.

Reporting for specific materials, systems and methods

We require information from authors about some types of materials, experimental systems and methods used in many studies. Here, indicate whether each material, system or method listed is relevant to your study. If you are not sure if a list item applies to your research, read the appropriate section before selecting a response.

Materials & experimental systems

n/a	Involved in the study	
☒	☐	Antibodies
☐	☒	Eukaryotic cell lines
☒	☐	Palaeontology and archaeology
☒	☐	Animals and other organisms
☒	☐	Human research participants
☒	☐	Clinical data
☒	☐	Dual use research of concern

Methods

n/a	Involved in the study	
☒	☐	ChIP-seq
☒	☐	Flow cytometry
☒	☐	MRI-based neuroimaging

Eukaryotic cell lines

Policy information about cell lines

Cell line source(s)	The Vero/hSLAM cell line is available from the European Culture Collection of Animal Cells, UK and BEI Resources, USA.
Authentication	Cells used were all genetically autheticated by ECACC
Mycoplasma contamination	All cells used were tested to ensure the absence of detectable mycoplasma

Commonly misidentified lines<br>(See ICLAC register)	None used
